# Additional sedative drugs to light sedation with dexmedetomidine is risk for delirium

**DOI:** 10.1186/2197-425X-3-S1-A332

**Published:** 2015-10-01

**Authors:** M Sakuraya, A Hirata, K Yoshida, N Kawamura, T Tsutsui

**Affiliations:** JA Hiroshima General Hospital, Critical Care Medicine, Hatsukaichi, Japan

## Introduction

Light sedation is preferred for critical ill patients. We often use dexmedetomidine for light sedation, but sometimes additional sedative drugs(such as midazolam and propofol) is needed. Benzodiazepine is risk for delirium, but it is unclear benzodiazepine use combined with dexmedetomidine is risk for delirium.

## Methods

This prospective observational study was conducted in open ICU, urban hospital(Hiroshima, Japan) between February 2013 and March 2015. Adult patients intubated within 24 hours from ICU admission and underwent light sedation with dexmedetomidine was eligible. Exclusion criteria were: age < 18, stroke, cardiopulmonary arrest, neurosurgery, seizure, traumatic brain injury. We compared alternative sedative dug use with no additional drug. Primary outcome is delirium incidence.

## Results

268 patients were eligible and 254 patients were analyzed. Median age (Q1-Q3) was 74 (66-81) years, 162 male(64%), median APACHE II score was 19 (15-23). 89 patients were administrated additional sedative drugs (midazolam 14, propofol 80). Delirium was observed more frequently compared with dexmedetomidine alone, 41 (46%) vs. 51 (31%), p = 0.02. Additional sedative drugs were associated with prolong ICU stay (5 (3-8) days vs. 3 (2-4.5) days, p < 0.001) and decreased ventilator free days (25 (21-26) days vs. 26 (26-27) days, p < 0.001).

## Conclusions

Additional sedative drugs to light sedation with dexmedetomidine might be risk for delirium.
